# Global Health Delivery 2.0: Using Open-Access Technologies for Transparency and Operations Research

**DOI:** 10.1371/journal.pmed.1000158

**Published:** 2009-12-01

**Authors:** Duncan Smith-Rohrberg Maru, Aditya Sharma, Jason Andrews, Sanjay Basu, Jhapat Thapa, Shefali Oza, Chhitij Bashyal, Bibhav Acharya, Ryan Schwarz

**Affiliations:** 1 Nyaya Health, Bayalpata Hospital, Ridikot VDC, Achham, Nepal; 2 Brigham and Women's Hospital, Department of Medicine, Boston, Massachusetts, United States of America; 3 Children's Hospital of Boston, Department of Medicine, Boston, Massachusetts, United States of America; 4 Contra Costa Regional Medical Center, Martinez, California, United States of America; 5 University of California San Francisco, Department of Medicine, San Francisco, California, United States of America; 6 San Francisco General Hospital, Division of Internal Medicine, San Francisco, California, United States of America; 7 Yale University School of Medicine, New Haven, Connecticut, United States of America

## Abstract

Duncan Maru and colleagues at Nyaya Health describe several simple Web 2.0 strategies they have implemented during the course of delivering medical and public health services in rural Nepal.

**Summary Points**

- The growing field of global health delivery is in need of technological strategies to improve transparency and operations research.
- Our organization has implemented several simple “Web 2.0” strategies while delivering medical and public health services in rural Nepal.
- These strategies help Nyaya Health improve transparency, receive critical commentary from outside experts, and compare approaches to organizing budgets, pharmaceutical procurement, medical treatment protocols, and public health programs.
- The platforms include quantitative outcomes data and logistics protocols on a wiki; an open-access, online deidentified patient database; geospatial data analysis through real-time maps; a blog; and a public line-by-line online budget.

## Introduction

Unprecedented resources have been mobilized for delivering health services in resource-limited areas over the last decade. The field of global health delivery aims to harness new finances, technical expertise, and political will to develop effective and efficient health systems throughout the world [1],[2]. A fundamental problem faced by practitioners in this field is to determine what strategies work best for delivering high-quality medical and public health services in different socioeconomic and political environments. The multiple stakeholders involved—local and national governments, nonprofit organizations, and private-sector businesses—have often failed to participate effectively and transparently in global health care delivery.

The 2008 Global Accountability Report (GAR), published by the organization One World Trust, revealed that some of the world's largest nonprofit organizations (including health care delivery organizations) scored worse on accountability measures than private, for-profit multinational corporations [3]. The GAR assesses organizations on four domains of accountability: transparency, participation, evaluation, and responsiveness. Only one of the nongovernmental organizations in the report met the One World Trust's basic minimum standard of accountability (a score of 70 out of 100). Transparency scores among nongovernmental organizations delivering health services were as low as 15 out of 100.

This lack of transparency not only reduces the accountability of individual programs,, it also misses an opportunity to advance global health delivery by establishing best practices in the field. Developing countries have long suffered from a paucity of comprehensive data on public health program impact [4]–[6]. The reliability of existing data is often in doubt, particularly as global funding mechanisms provide incentives for misreporting [7]. Even more sparse are data on diagnostic, treatment, and organizational practices in the delivery of primary care in resource-limited settings [8]. Just as clinical trial and genomic data have been “open sourced,” leading to new advances in biomedicine [9],[10], data made public by networks of medical providers and public health practitioners can be useful for establishing standards and methods for care delivery and public health practice [11],[12].

## Strategies for Global Health Delivery 2.0

One strategy for creating such a system among global health programs is to provide open access to accurate and up-to-date information online. “Web 2.0” technologies—software that allows for rapid, Internet-based collaboration among multiple users—can improve transparency among organizations participating in global health delivery. These have recently been deployed extensively in resource-rich areas [13],[14], but have not been implemented widely in resource-limited settings. Here, we provide an overview of several of the tools that our organization, Nyaya Health, has been implementing to improve transparency, receive critical commentary from outside experts, and compare approaches to organizing budgets, pharmaceutical procurement, medical treatment protocols, and public health programs.

Our organization, run by US- and Nepal-based health professionals, operates a health center in the district of Achham, Nepal, one of the most remote and impoverished communities in South Asia. The district, just emerging from a decade-long civil war, has minimal health infrastructure: there were no physicians for a population of 250,000 people prior to our construction of a regional health center [15]. Owing to the telecommunications challenges in Achham, Nyaya Health has developed strategies that require minimal bandwidth and computing infrastructure. Our work has been powered by simple, free, easy-to-learn systems that are enabled by the open and collaborative nature of the Web 2.0 strategy.

Nyaya Health utilizes five main Web 2.0 strategies to share its operations protocols, outcomes data, costs, and organizational processes (Box 1): quantitative outcomes data and logistics protocols on a wiki (a Web site that allows multiple users to quickly edit pages); an online, open-access, deidentified patient database; geospatial data analysis through real-time maps (electronic geographical information systems); qualitative information in the form of prose reports describing patients, logistics, management, and community politics on a blog (a website that displays email postings by date); and a public line-by-line online budget. In each of these endeavors, transparency and operations research go hand in hand. By maintaining all of our operational research data in a public online forum, we are able to effectively communicate our continued process of programmatic revision and improvement, achieving institutional memory and acquiring critical feedback from our colleagues and supporters.

**Box 1. Technological Platforms for Global Health Delivery 2.0**

*Definitions provided are specific to how these technologies are used for global health delivery.*

**Wiki**

A wiki is an online, open-access portal of protocols and data describing health care delivery programs. A wiki can be used to share detailed clinical and operational information and critical reviews of services through online spreadsheets and graphs. Example: http://wiki.nyayahealth.org/.

**Aggregate patient databases**

Deidentified, up-to-date aggregate patient data can be input into charts and graphs for review and research. These databases describe the outcomes data necessary for rigorous monitoring and evaluation of global health delivery programs. Example: http://wiki.nyayahealth.org/PharmacyData.

**Spatial maps**

These maps provide dynamic spatial information about service utilization, to assist in program planning and responses to emerging health problems across borders. They can describe where health care is accessible, where it is not, and where future services should be located. Examples: http://healthmap.org [21], http://wiki.nyayahealth.org/SpatialMapping.

**Blog**

A blog is an online repository of narrative descriptions of patients, logistics, management, and local socioeconomics or politics. A blog can help describe how global health delivery works, or fails to work, in accessible and personal language. Examples: http://msf.ca/blogs, http://globalhealthdelivery.org/.

**Detailed budgets**

Disaggregated budget details are critical for internal quality control, for external transparency and accountability, and for collaboration. These budgets describe the expenditures and material inputs for health services. Example: http://wiki.nyayahealth.org/Budget.

The wiki provides an indexed, tagged repository of clinical protocols, management strategies, programmatic work plans, and clinical engineering details in real time, as they are developed, improved upon, and expanded [16]. This serves as a publicly accessible “field manual” pertaining to critical aspects of global health delivery that are not typically available in public health textbooks, and which require experience to describe in detail. For example, we describe how to estimate the energy needed by a primary care center laboratory and how to appropriately connect different electrical components and backup generators in a manner that is reliable for rural health clinics. The wiki page is easily editable by staff and volunteers, which facilitates efficient collaboration on new programs as we develop strategies and work plans. As an example, we have successfully used the wiki to develop a locally appropriate malnutrition protocol with collaborating experts in dialogue with our on-site personnel. Changes to the wiki are seen immediately, allowing for efficient and productive dialogues about programs. This allows for real-time collaboration on programs as they evolve to meet the changing needs of communities. While editing is restricted to Nyaya Health collaborators only, all pages are viewable to the public so that outside colleagues can also comment upon and utilize these pages.

**Figure 1:**
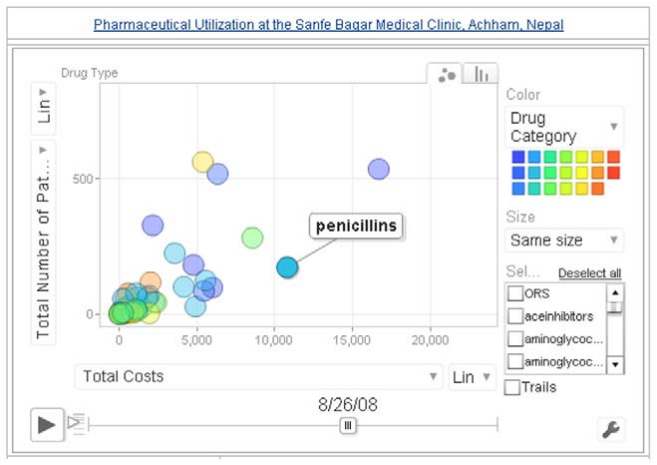
Screenshot of our pharmaceutical tracking system. Gapminder's (http://www.gapminder.org) free software provides an intuitive interface for depicting utilization patterns of key pharmaceutical drugs and categories that is published regularly to the web.

Aggregated, deidentified, online, public access databases are also an important aspect of accountability and, with proper standardization, can greatly improve accountability in global health practice. Nyaya Health's strategy for data input, presentation, and monitoring involves local data entry, processing, and posting of these data in the form of online tables, charts, and graphs of both classical epidemiological indicators and newer social indicators to evaluate the social equity of our programs [17]. These data enable easy, frequent review of clinical programs for planning, revising, and reshaping clinical practices. Online “widgets” (easily created graphics for displaying data [Figure 1]), allow us to view the number of patients and costs associated with different pharmaceutical classes over time. By making the aggregate data available online, we can facilitate research and collaboration on strategies for pharmaceutical procurement and prescription in health care delivery programs. By reviewing data from provider prescribing patterns, we have been able to identify issues for which explicit clinical protocols were needed, such as management of dyspepsia and treatment of chronic obstructive pulmonary disease. The next step will be to utilize these data to determine whether the protocols effectively change prescribing practices. Furthermore, in the long run and with sufficient standardization, publishing open-access, easily updated data in a standard manner can help detect disease outbreaks and mobilize international responses to public health problems. Ensuring the privacy and anonymity of patient information is essential, and we take many measures to this end, including careful deidentification of patient records, use of secure servers, and physical locks of medical records in the clinic.

**Figure 2:**
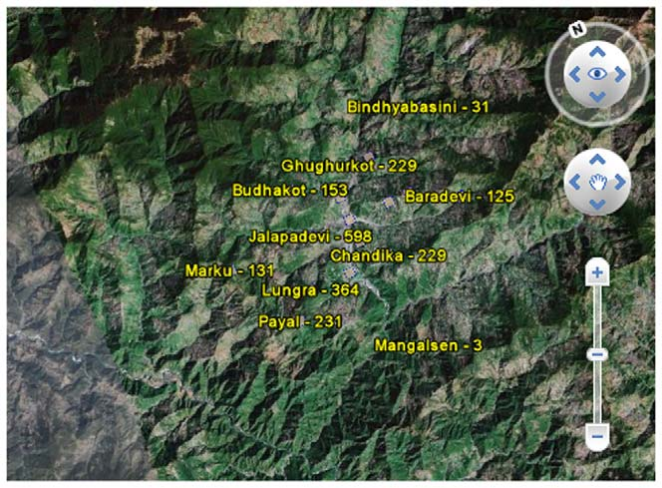
Screenshot of Nyaya Health mapping project. This particular image shows the number of patients from select surrounding villages over a six-month period. Using readily available GPS mapping information and data from our electronic patient database, we can map out service utilization and access to medical care. This helps in planning the geographic aspects of our community health programs.

Online, dynamic, publicly accessible maps of local health services (Figure 2) can play an important role in the planning of public health programs and medical services [18]. The main data points for Nyaya Health's map are taken from the geographical coordinates of patients' home villages and correlated with clinical data. The map improves the logistics of planning the locations and timing of mobile health service delivery (via community health care workers) by tracking pharmaceutical utilization and diagnoses among local villages. Eventually, we hope to implement “syndromic surveillance” by mapping symptoms and diagnoses to rapidly identify emerging changes in local epidemiological conditions.

The blog serves as a forum for discussing relevant deidentified patient cases, clinical operations details, and organizational challenges and successes [19]. Staff and volunteers write stories and post images. This provides opportunities to discuss critical logistical issues that cannot easily be captured in quantitative forms, particularly when they require discussion of socioeconomic and political issues. Patient stories are deidentified, including all clinical images. Donors, personnel, and the general public can read the blog to develop a more realistic sense of the process of improving health infrastructure and delivering services in a difficult environment. Other colleagues involved in global health delivery can use these experiences to guide their own work or to post comments that provide us with useful suggestions for improvement.

**Figure 3:**
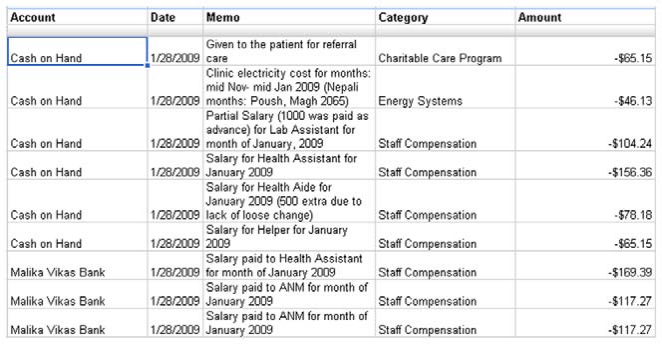
Screenshot of the Nyaya Health budget. The line-by-line budget, updated monthly, is posted online. The dollar amounts shown are converted from Nepali Rupees using daily closing exchange rates.

Finally, publicly available line-by-line budgets can play a critical role in improving the financial aspects of global health delivery [20]. The raw inputs to these expenditure displays are entries from the local accounting system, which includes everything from major purchases like generators and oxygen concentrators to small items like food for staff meetings. These data are presented through standardized online spreadsheets that allow donors, collaborators, and staff to easily access financial information (Figure 3). These data also help other practitioners to plan health delivery by seeing the breakdown in our expenditures alongside data such as population served, disease burden, and services rolled out.

## Discussion

There are several important challenges and limitations to our use of Web 2.0 strategies. The approach discussed here is not yet accessible to many of our patients and staff, who are for the most part not computer literate. Establishing a reliable internet connection itself is costly in many rural areas; Nyaya Health has invested heavily in telecommunications to overcome the lack of infrastructure. This has included investments in hardware such as a satellite dish and computers (all of which have been donated from supporters), as well as software to prevent viruses and other malware from affecting security and performance. Interestingly, the technical aspects of actually deploying the software were not particularly challenging. It has been critical, however, that our leadership staff includes several individuals with epidemiological and data management experience.

Achieving sufficient engagement with local staff has been a challenge and has required us to identify improvements to staff contracts and incentive systems. Belief in the utility of data and use of evidence to drive health care is a cultural shift that is challenging to enact, whether in the United States or in Nepal. The demands of high patient volumes compete with the demands of data collection in the minds of providers. Nyaya Health has taken the policy of mandating monthly clinical data (on patients seen, pharmaceuticals used, money spent, types of cases, and care provided) reviews of clinical data tied in with very similar monthly data reports due to the government. Since the government reports are labour-intensive to write, having these reports generated electronically has provided a clear benefit to staff. Monthly reviews and analysis, with posting on the public Web sites, are required in any case to ensure that data are being collected properly, to receive rapid and useful feedback on services, and to identify any gaps in our data system.

These tools should not be confused with transparency and accountability structures at a local level. There is no replacement for community oversight and effective participation of patients in the design and implementation of their own public health systems. The community members who receive our care are not principally concerned with Web 2.0 applications. Still, effective delivery of care to these communities may be facilitated by the use of such technologies, especially as efforts expand to increase access to computer hardware and education.

The power of open-access, Web 2.0 applications will continue to grow, but a critical question is how best to deploy these technologies and evaluate their impact. The costs of these strategies can be minimized through the use of publicly available software programs that are accessible to nonspecialist analysts. The most important factor in implementation is less a matter of financial resources than one of fostering an ethic within health delivery organizations that data must be rigorously collected and published in a public and accessible format. Over the next several years, we hope that more organizations develop and test these tools to share their experience, data, and institutional knowledge in the effective delivery of health services in resource-denied areas. Evaluation metrics need to be developed to assess the impact of these strategies on clinical outcomes, costs, staff and patient satisfaction, and responsiveness to outside criticism and community demands. Developing common standards will improve clinical effectiveness and resource allocation to build a truly rigorous and innovative science of global health delivery.

## Abbreviations

GAR: Global Accountability Report
